# Cell therapy for scleroderma: progress in mesenchymal stem cells and CAR-T treatment

**DOI:** 10.3389/fmed.2024.1530887

**Published:** 2025-01-15

**Authors:** Liting Chen, Rongshan Huang, Chaoshuo Huang, Guiming Nong, Yuanyuan Mo, Lvyin Ye, Kunhong Lin, Anping Chen

**Affiliations:** ^1^Department of Rheumatology and Immunology, Qinzhou First People's Hospital, Qinzhou, Guangxi, China; ^2^Minda Hospital of Hubei Minzu University, Enshi, China

**Keywords:** scleroderma, cell therapy, mesenchymal stem cells, mesenchymal stem cell exosomes, chimeric antigen receptor T cells

## Abstract

Cell therapy is an emerging strategy for precision treatment of scleroderma. This review systematically summarizes the research progress of mesenchymal stem cell (MSC) and chimeric antigen receptor T cell (CAR-T) therapies in scleroderma and discusses the challenges and future directions for development. MSCs possess multiple functions, including immunomodulation, anti-fibrosis, and promotion of vascular regeneration, all of which can improve multiple pathological processes associated with scleroderma. Studies have demonstrated that MSCs can alleviate skin fibrosis by inhibiting CCL2 production and reducing the recruitment of pathological macrophages; their paracrine effects can exert extensive regulatory functions. CAR-T cell therapy ca specifically target and eliminate autoreactive immune cells, exhibiting enhanced specificity and personalized potential. Different cell therapies may have complementary and synergistic effects in treating scleroderma, such as MSCs exerting their effects through paracrine mechanisms while CAR-T cells specifically eliminate pathological cells. Furthermore, cell-free therapies derived from MSCs, such as extracellular vesicles or exosomes, may help circumvent the limitations of MSC therapy. Although cell therapy has opened new avenues for the precision treatment of scleroderma, it still faces numerous challenges. In the future, it is essential to strengthen integration of basic and clinical research, establish standardized protocols for cell preparation and quality control, develop personalized treatment plans, and rationally combine cell therapy with existing treatment methods to maximize its advantages and improve patient prognosis and quality of life.

## 1 Introduction

Scleroderma is a chronic autoimmune disease characterized by widespread microvascular damage, endothelial cell injury, immune activation, and fibrosis of the skin and internal organs (1). Patients typically present with skin hardening, thickening, facial changes, and digital ulcers. In severe cases, vital organs such as the lungs, kidneys, and heart may be involved, threatening life (2). Although the exact cause of scleroderma remains unclear, it is widely believed that its pathogenesis is closely related to dysfunction in the vascular and immune system, with sustained immune activation and uncontrolled fibrosis playing key roles in disease progression (3). This suggests that when exploring treatment strategies for scleroderma, we should focus on suppressing abnormal immune responses and fibrotic processes.

Treatment of scleroderma still faces many challenges. For localized scleroderma, mainly local and physical therapies utilized, but their efficacy is limited. In the case of diffuse scleroderma, immunosuppressants such as glucocorticoids and cyclophosphamide can control the disease to some extent; however, their effectiveness is often inadequate, and the efficacy associated side effects can be significant, making it difficult to halt disease progression fundamentally. The effects of vasodilators and antifibrotic agents are also unsatisfactory. As such, traditional treatments struggle to meet the clinical needs of scleroderma patients, highlighting the urgent need for the development of new strategies to improve prognosis. This highlights the important research significance of cell therapy in the field of scleroderma.

Stem cells have become ideal tools for treating various diseases due to their self-renewal and multi-lineage differentiation potential. In recent years, stem cell therapy has shown good prospects in clinical studies of autoimmune diseases such as systemic lupus erythematosus and rheumatoid arthritis (4), providing inspiration for its application in scleroderma treatment. As a class of abundantly sourced and easily isolated adult stem cells, mesenchymal stem cells (MSCs) have garnered significant attention for their low immunogenicity, immunomodulation, and tissue repair properties (5, 6). Previous studies have shown that MSCs can exert anti-scleroderma effects through the paracrine secretion of cytokines, extracellular vesicles, and other products at multiple levels, including regulating immune responses, improving fibrosis, and promoting damaged tissue repair (7). This suggests that MSCs may become a new cellular tool for scleroderma treatment. In addition, chimeric antigen receptor T cell (CAR-T) therapy, an emerging immune cell technology that has made breakthroughs in B-cell malignancy in recent years, may also be applied to autoimmune disease treatment by genetically engineering T cells to specifically target and kill cells (8). Given the immune dysregulation characteristics of scleroderma, whether CAR-T therapy to provide new breakthroughs in its treatment warrants in-depth exploration.

## 2 Research progress of mesenchymal stem cell therapy for scleroderma

### 2.1 Biological characteristics and mechanisms of action of mesenchymal stem cells

Mesenchymal stem cells (MSCs) are a type of adult stem cell characterized by their self-renewal and multi-lineage differentiation potential. Initially discovered in bone marrow, subsequent studies showed their widespread presence in adipose tissue, umbilical cord, and various other tissues (6, 9–11). Morphologically, MSCs are spindle-shaped or fibroblast-like, expressing specific surface markers such as CD105, CD73, and CD90, but not express hematopoietic and endothelial cell markers like CD45, CD34, CD14, CD11b, CD79α, CD19, and HLA-DR (6, 9, 10). MSCs can differentiate into osteoblasts, chondrocytes, adipocytes, and other cell lineages, thereby playing a key role in tissue repair and regeneration (11).

In recent years, numerous studies have revealed the unique advantages of MSCs in regulating immune responses, inhibiting fibrosis, and promoting angiogenesis, making them as a focus in autoimmune disease treatment (12–16). Mechanistic studies indicate that MSCs can secrete various immunosuppressive factors such as IL-10, HGF, and PGE2 to inhibit the proliferation and activation of immune cells like T cells, B cells, NK cells, and dendritic cells. This process induces inducing immune tolerance and thus alleviating autoimmune damage (15, 17, 18). Moreover, MSCs can downregulate the expression of pro-fibrotic factors such as TGF-*β* and CTGF to inhibit fibroblast proliferation and collagen deposition, thereby reducing tissue fibrosis (12, 19, 20). Simultaneously, MSCs secrete angiogenic factors like VEGF and HGF to stimulate endothelial cell proliferation and angiogenesis, thereby improving tissue ischemia and damage (13, 18). Given the synergistic effects of MSCs in immunomodulation, anti-fibrosis, and tissue repair, they may become an ideal cell therapy approach to intervene in complex pathological processes such as scleroderma.

**Figure 1:**
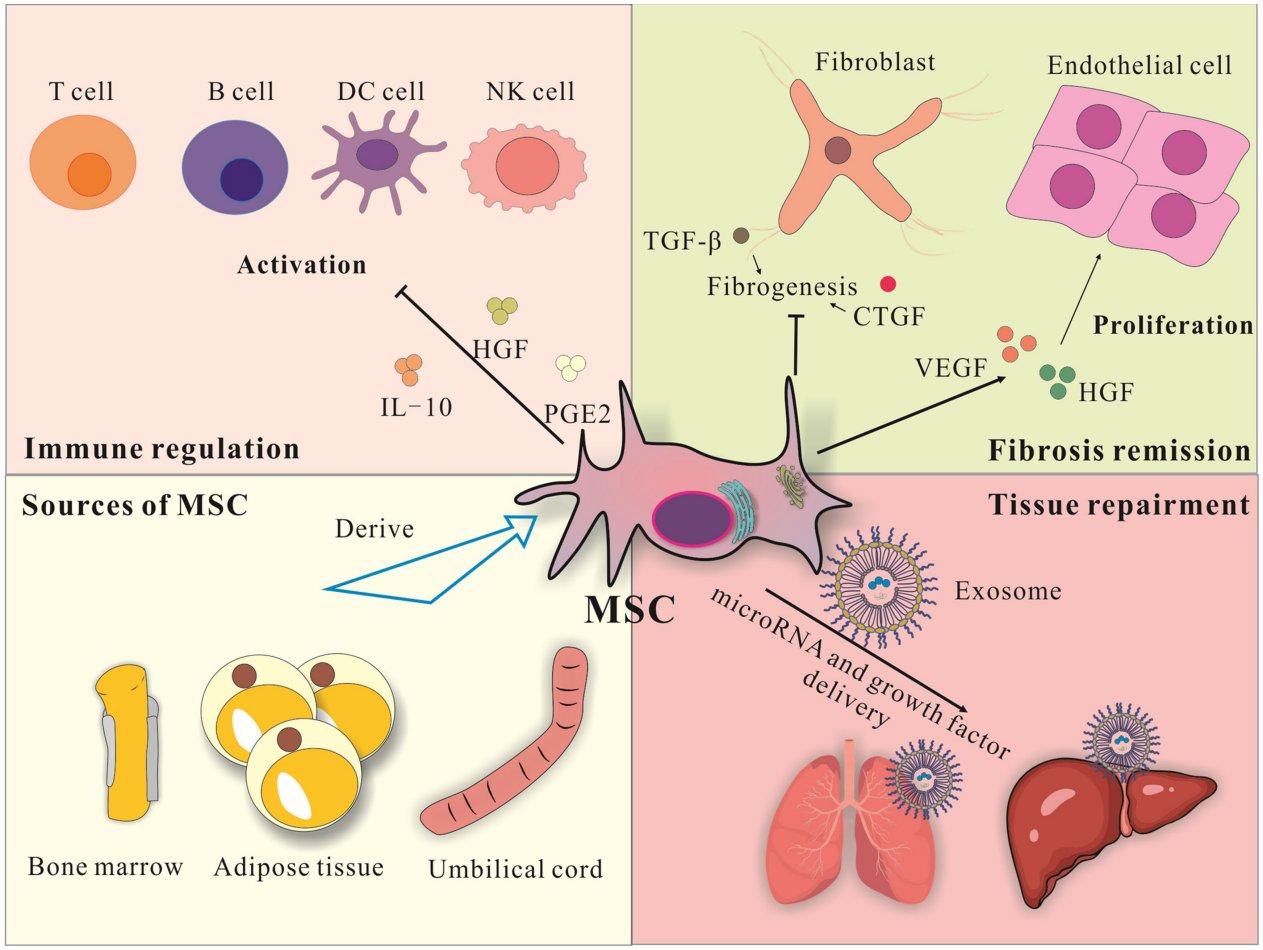
Illustration of Application of CAR-T Therapy treatment. Mesenchymal stem cells can be derived from bone marrow, fat and umbilical cord. These stem cells can themselves home to diseased tissues, or release vesicles or exosomes carrying various signaling substances such as proteins, nucleic acids and micro-RNAs through paracrine action to act on diseased tissues. The main affected organs in systemic sclerosis include the skin, lungs, kidneys, heart and blood vessels. Stem cells themselves or the exosomes secreted by them can act on the diseased tissues of systemic sclerosis and improve the inflammation and fibrosis of these tissues.

Although the exact mechanisms by which MSCs exert their therapeutic effects are not fully elucidated, studies have suggested their paracrine effects may be key. Extracellular vesicles, especially exosomes, secreted by MSCs can selectively enrich and deliver various bioactive substances such as, microRNAs and growth factors to damaged tissues and effector cells, regulating gene expression and conveying the therapeutic effects of MSCs (21, 22). This discovery provides new ideas for revealing the mechanisms of action of MSCs and optimizing MSC treatment strategies. Future studies should explore the component characteristics and functional regulatory networks of MSC exosomes at both the molecular and cellular levels to clarify their therapeutic mechanisms in diseases like scleroderma, thereby providing a theoretical guidance and material basis for cell therapy (Figure 1).

### 2.2 Therapeutic effects of mesenchymal stem cells in scleroderma animal models

#### 2.2.1 Comparison of efficacy of mesenchymal stem cells from different sources in scleroderma animal models

To explore the application prospects of MSCs in scleroderma treatment, several studies have employed mouse models induced by bleomycin or hypochlorous acid to investigate the therapeutic effects of MSCs from bone marrow, adipose tissue, umbilical cord, and other tissue sources (11, 23–28). Zhang et al. compared the efficacy of single tail vein injection of autologous or allogeneic bone marrow MSCs derived from the mouse in a bleomycin-induced scleroderma mouse model. They found that both sources of MSCs effectively alleviated inflammatory infiltration and collagen deposition in skin and lung tissues, reduced the imbalance of Th1/Th2 cell cytokines in the lungs, and have similar efficacy (26). Abdel et al. compared the therapeutic effects of allogeneic bone marrow MSCs and adipose MSCs on a hypochlorous acid-induced scleroderma mouse model. The results showed that both types of MSCs improved skin and lung fibrosis; however, adipose-derived MSCs were superior to bone marrow-derived MSCs in reducing inflammatory cytokines and autoantibody levels (17). In addition, Liang et al. confirmed that human umbilical cord MSCs could dose-dependently inhibit skin thickening, inflammation, and collagen deposition in bleomycin-induced scleroderma mice, with high-dose (1 × 10^6 cells/mouse) being more effective than medium and low doses (2.5 × 10^5 and 5 × 10^5 cells/mouse) (28).

However, the aforementioned studies mainly used intravenous administration, and it is unclear whether MSCs can effectively home to damaged tissues. Considering that scleroderma lesions mainly involve local tissues such as the skin and lungs, future studies should explore more precise cell delivery strategies such as local administration, to enhance the enrichment and therapeutic efficacy of MSCs in lesion tissues. In addition, scleroderma animal models differ considerably from clinical phenotypes, and caution is needed when extrapolating the above results to the clinic. Translational research based on humanized animal models and clinical samples to provide reliable evidence for formulating optimal MSC treatment regimens.

#### 2.2.2 Mechanism exploration of mesenchymal stem cell exosomes exerting effects through MicroRNAs and signaling pathways

The paracrine effects of MSCs are an important mechanism through which they exert their therapeutic effects. Among them, extracellular vesicles, especially exosomes, secreted by MSCs, have received much attention for their potential role in scleroderma treatment. Studies have shown that MSC-derived exosomes can selectively enrich and deliver various bioactive substances such as microRNAs and growth factors, to regulate gene expression and signal transduction in recipient cells, conveying the therapeutic effects of MSCs (21, 22). Yamashita et al. isolated and purified human MSC exosomes and administered them in bleomycin-induced scleroderma mice. The results demonstrated that exosomes significantly alleviated fibrosis in the skin and visceral organs and inhibited fibroblast activation and macrophage infiltration. Further mechanistic studies revealed that MSC exosomes were enriched in anti-fibrotic microRNAs such as miR-196a, which could target and downregulate the expression of fibrotic genes like Col1a1, Col1a2, and αSMA in fibroblasts (22). Similarly, Zhang et al. also confirmed that MSC exosomes alleviated skin hardening and lung fibrosis in scleroderma mice by carrying miR-29a, which inhibits the fibrosis-related TGF-*β*/Smad signaling pathway (21).

These studies reveal, at the molecular level, that MSC exosomes sever as important carriers of MSCs’ therapeutic effects, exert anti-fibrotic effects by selectively enriching and delivering specific bioactive substances, such as microRNAs, to regulate fibroblast phenotype and collagen metabolism. This provides new ideas for elucidating the mechanisms of action of MSCs and optimizing MSC treatment strategies. Given the complex composition and diverse structures and functions of MSC exosomes, future studies should explore their component characteristics and functional regulatory networks to establish standardized systems for exosome preparation and quality control, laying the foundation for advancing the clinical application of MSC exosomes. In addition, the mechanisms by which exosomes achieve tissue-specific enrichment and targeted delivery still unclear, limiting their therapeutic efficacy. In-depth exploration of the biodistribution patterns and targeting mechanisms of exosomes and optimization of administration routes and dosing regimens will help improve the therapeutic effects and clinical translation potential of exosomes. In summary, as a new cellular therapy medium, MSC exosomes may overcome the bottlenecks faced by MSC treatment of scleroderma, but transforming them into a mature treatment modality still requires extensive in-depth research.

### 2.3 Current status of clinical research on mesenchymal stem cell therapy for scleroderma

Given the therapeutic potential demonstrated by MSCs in scleroderma animal models, several preclinical studies have conducted preliminary explorations in scleroderma patients, using autologous or allogeneic MSC infusion to assess their safety and efficacy (29–36). The majority of participants were patients with diffuse scleroderma characterized by extensive skin and visceral involvement. They were given single or multiple intravenous infusions of bone marrow or umbilical cord-derived MSCs at a dose of (1, 2) × 10^6/kg, with efficacy assessed at 3–12 months of follow-up. The main observation indicators included skin hardening improvement (modified Rodnan skin score, mRSS), vascular lesion manifestations (digital ulcers, Raynaud’s phenomenon), lung function indicators (forced vital capacity FVC, lung diffusion function DLCO), autoantibody titers (14, 20, 32, 33). Safety evaluation mainly observed the occurrence of infusion-related adverse reactions and complications, such as infections. Most studies found that 4–12 weeks post-MSC infusion, the degree of skin hardening in the face, hands, and trunk was significantly reduced, skin luster and elasticity improved, and mRSS scores decreased by more than 30% from baseline (6, 14, 37, 38). At the same time, MSC therapy was found to promote the healing of refractory digital ulcers, relieve pain, and improve patients’ quality of life (14, 18). For patients with concomitant pulmonary hypertension, right heart function improved, and pulmonary artery pressure decreased after MSC treatment (6). In addition, MSCs could also alleviate visceral involvement such as gastrointestinal motility disorders (18). These studies indicate that intravenous infusion of MSCs can comprehensively improve skin, vascular, and visceral lesions in scleroderma patients, with preliminary but encouraging efficacy.

Existing research indicates that MSC therapy is well-tolerated, with only a few patients experiencing transient mild infusion reactions, such as fever and rash, and no reports of serious adverse events (20, 33). In terms of efficacy, most studies have observed significant reductions in skin hardening and mRSS scores after MSC treatment, with effects lasting for several months (29, 32, 33, 36). Some patients with concomitant interstitial lung disease also showed improvements in lung function indicators and CT imaging (31, 33). In addition, serum levels of inflammatory cytokines, such as IL-6 and TNF-*α* decreased after MSC treatment, as did titers of autoantibodies such as anti-Scl-70 and anti-centromere antibodies (20, 36). The above studies preliminarily confirmed the safety and potential efficacy of MSC therapy for refractory scleroderma patients, but small sample sizes, lacked control groups, and efficacy evaluation indicators and follow-up times were not uniform enough. On the other hand, existing studies are mostly single-center, open-label clinical trials lacking uniform inclusion criteria, administration regimens, and efficacy judgment standards, making objective comparisons and meta-analyses difficult. In the future, evidence-based guidelines for scleroderma cell therapy should be developed to standardize clinical trial design, unify efficacy judgment and adverse event reporting standards, and improve the comparability and evidence level of different study results. At the same time, stratified research and subgroup analyzes should be conducted for patients with disease stages and clinical manifestations to clarify the optimal indications and timing of MSC therapy and achieve precision treatment. Currently, clinical research on MSC therapy for scleroderma is still in its infancy, and many key scientific questions await systematic in-depth exploration, such as the *in vivo* homing distribution and pharmacokinetic characteristics of MSCs, optimal administration routes, dosage regimens, and treatment courses, and optimization of combination therapy regimens. Large-sample, multicenter prospective cohort studies are urgently needed to accumulate high-quality evidence-based medicine evidence.

Although clinical research on MSC therapy for scleroderma has made positive progress, transforming it into a mature treatment strategy still faces many challenges. The primary issue is the standardization of MSC preparation and quality control. Uniform protocols must be established for MSC sourcing, isolation, purification, and *in vitro* expansion to preferentially select low-immunogenicity allogeneic sources, such as umbilical cord tissue, and to ensure the stability and comparability of preparation quality. Secondly, the allogeneic MSC therapy has long-term immunogenicity and tumorigenicity risks still require vigilance (20, 39). Furthermore, whether intravenous infusion can deliver MSCs to lesion tissues remains questionable, and optimal administration routes and dosage regimens need further optimization (5). In addition, to objectively evaluate efficacy, a unified evaluation indicator system needs to be established, taking into account improvements in patient symptoms, signs, and histopathology at multiple levels (39).

## 3 Application of adipose-derived mesenchymal stem cells and tissue engineering in scleroderma treatment

Adipose tissue serves as an important source of MSCs, and ADSCs have the advantages of convenient material acquisition, minimal trauma, and high yield. Compared to bone marrow-derived MSCs, ADSCs have stronger proliferative capacity, differentiation potential, and immunomodulatory effects, and they are less age-dependent (11, 40, 41). In addition to osteogenic, chondrogenic, and adipogenic lineages, ADSCs can also differentiate into endothelial cells, smooth muscle cells, and other cell types. Furthermore, they secrete angiogenic factors such as VEGF and HGF, which play an important role in tissue repair (11, 42).

For autoimmune diseases such as scleroderma, ADSCs offer distinct advantages in immunomodulation and anti-fibrosis (11, 40, 43). ADSCs derived from scleroderma patients express more pluripotency genes such as IDO-1 and SOX2, have stronger ability to secrete cytokines such as IL-6 and HGF, and have more significant inhibitory effects on T cells (41, 44). Animal experiments have confirmed that the transplantation of allogeneic or autologous ADSCs can effectively improve skin and lung fibrosis in hypochlorous acid-induced mice, with efficacy comparable to bone marrow MSCs, but ADSCs have greater advantages in reducing inflammatory cytokines such as TNF-*α* and IL-1β and increasing the MMP-1/TIMP-1 ratio (40). In addition, extracellular vesicles secreted by ADSCs have regulatory effects on fibroblasts and can reverse their pro-fibrotic phenotype (44). Therefore, ADSCs may become an ideal choice for cell therapy in scleroderma.

Given the therapeutic effects of ADSCs in scleroderma animal models, adipose tissue and ADSC transplantation strategies have begun to be explored clinically (11, 45, 46). For facial skin hardening, autologous fat transplantation is a minimally invasive and individualized treatment method that can not only improve skin texture and contour but also locally deliver ADSCs to exert immunomodulatory effects and promote microcirculation reconstruction (11, 45). In the case of digital ulcers, local multiple injections of autologous adipose tissue extract can significantly promote wound healing and improve pain and function (42, 45). A prospective study conducted 6 months of autologous fat transplantation treatment for 13 patients with localized scleroderma and digital ulcers showed that all ulcers healed, pain was relieved, quality of life improved, and no recurrence was observed at 1-year follow-up (45). For diffuse scleroderma with interstitial lung disease, bronchial injection of autologous adipose tissue extract can safely and effectively alleviate pulmonary inflammation and delay the progression of pulmonary fibrosis (11). However, current studies have small sample sizes, lack control groups and uniform efficacy evaluation standards, and still require large-scale randomized controlled trials to further verify their long-term efficacy and safety.

## 4 Research progress of chimeric antigen receptor T cell (CAR-T) therapy for scleroderma

### 4.1 Basic principles of CAR-T cell therapy and its application in autoimmune diseases

In recent years, chimeric antigen receptor T cell (CAR-T) therapy, as a major breakthrough in the field of cancer immunotherapy, has brought new hope to numerous patients with refractory malignancies. CAR-T cells are T lymphocytes that have been genetically engineered to specifically recognize and kill tumor cells (45). Unlike conventional T cell receptors (TCRs), CARs can directly recognize tumor cell surface antigens in a non-MHC-restricted manner and release perforin and granzyme B to induce apoptosis in tumor cells. This mechanism demonstrates enhanced killing efficiency and broader application prospects (47, 48). CD19-targeted CAR-T cells have made breakthrough progress in the treatment of B-cell malignancies, with multiple clinical studies showing that CD19 CAR-T cells can induce complete remission in up to 80–90% of patients with refractory or relapsed B-cell leukemia and lymphoma (49). These encouraging efficacy results provide new ideas and directions for CAR-T cell therapy in autoimmune diseases.

Given the key role of B cells in the pathogenesis of autoimmune diseases, CD19 CAR-T cells may effectively control disease progression by specifically clearing autoreactive B cells, blocking autoantibody production, and reestablishing immune tolerance (50, 51). Compared to the B-cell- depleting drug rituximab currently approved for autoimmune diseases, CAR-T cell therapy has several advantages: first, CAR-T cells have higher killing efficiency and can clear deep-tissue B cells to maintain long-term efficacy; second, CAR-T cells have *in vivo* proliferative capacity and can exert sustained antitumor effects following a single infusion; third, CAR-T cells also have multiple immunomodulatory effects such as regulating T cell subset balance and inducing regulatory T cells (Tregs) (4, 45, 47). Therefore, CD19 CAR-T cells may become an innovative treatment option for autoimmune diseases, particularly those that are mediated by B cells.

CAR-T therapy in the field of autoimmune diseases is still in its infancy, but it has shown good application prospects in refractory rheumatic immune diseases such as systemic sclerosis (SSc). As an acquired autoimmune disease characterized by extensive skin and visceral fibrosis, SSc seriously affects patient survival and quality of life, and conventional immunosuppressive therapy has poor efficacy. Research indicates that chimeric antigen receptor technology provides new ways to overcome the limitations of conventional SSc treatment. CAR-T cells designed to target B cell subsets and SSc-specific antigens may play unique advantages in blocking SSc autoimmune responses and reversing fibrotic processes. This article will focus on discussing the research progress of CAR-T cell therapy in SSc and related rheumatic immune diseases, analyzing the opportunities and challenges it faces, and looking forward to its future development direction.

### 4.2 Current status of CD19-CAR-T cell therapy for systemic sclerosis and autoimmune disease

The results of multiple preclinical and early clinical studies support the feasibility and potential benefits of using CD19 CAR-T cells for the treatment of autoimmune diseases, such as SSc. Ingelfinger et al. first reported a case of a refractory SSc patient who received HLA-mismatched donor-derived CD19 CAR-T cell infusion after conventional immunosuppressive therapy had failed. The patient’s skin hardening and lung function improved significantly, and no serious adverse reactions occurred, suggesting that CD19 CAR-T cells are both safe and effective for SSc (52). A further multicenter phase I clinical trial enrolled 15 autoimmune disease patients, including SSc, systemic lupus erythematosus (SLE), and idiopathic inflammatory myopathy (IIM). Following a single CD19 CAR-T infusion treatment, the European Scleroderma Study Group (EUSTAR) activity scores of all SSc patients and disease indexes of SLE and IIM patients decreased significantly, with only a few mild cytokine release syndrome (CRS) occurrences, confirming the broad-spectrum anti-autoimmune effects and safety of this therapy. In addition to conventionally sourced CAR-T cells, healthy donor CAR-T cells can also be gene-edited and directly applied to patients as an “off-the-shelf” treatment product. The CRISPR/Cas9 was used to knock out the endogenous TCR and HLA-I genes of CAR-T cells to obtain universal CD19 CAR-T cells, which were infused into 1 patient with refractory necrotizing myopathy and 2 patients with diffuse SSc. At 6 months of follow-up, disease activity and organ function improved significantly, and no rejection reactions or severe CRS occurred, suggesting the safety and efficacy of gene-edited allogeneic CAR-T cells in SSc treatment (53). As research deepens, multiple centers at home and abroad are conducting prospective randomized controlled clinical trials to obtain more high-quality evidence-based medicine to support the application and promotion of CD19 CAR-T in the treatment of SSc and other rheumatic immune diseases (1, 54).

Existing research has preliminarily confirmed the feasibility and potential benefits of CD19 CAR-T cell therapy for autoimmune diseases such as SSc and inflammatory myopathy, but its clinical application still faces many challenges. For example, CAR-T therapy is expensive, and the economic burden on patients is heavy. Additionally, the long-term efficacy and safety are still unclear, and there may be risks of disease recurrence and secondary infection complications; its drug dosage, infusion frequency, combination therapy regimen. Furthermore, the monitoring and management processes for adverse reactions still need further improvement. Therefore, large-sample, multicenter prospective randomized controlled studies are still needed in the future to comprehensively evaluate the benefits and risks of CAR-T therapy in the long-term management of SSc and related rheumatic immune diseases. These studies should aim to establish standardized treatment regimens and adverse reaction prevention and management of adverse reactions, ultimately transforming this emerging technology into a routine clinical treatment to benefit a larger patient population.

### 4.3 Exploration of CAR-T cell therapy optimization strategies for scleroderma

Although CD19 CAR-T treatment has shown feasibility and potential efficacy in SSc, how to further improve CAR-T cell function, reduce adverse reactions, prolong the duration of efficacy, and develop it as a routine treatment for SSc are still key issues that need to be urgently addressed (55, 56).

The optimized design of next-generation CAR structures may improve the anti-tumor activity and persistence of CAR-T cells. Third-generation CARs introduce two or more co-stimulatory domains that can more effectively activate T cells, promote their proliferation and cytokine secretion, and enhance their cytotoxic capabilities and *in vivo* persistence. Hunder et al. used this strategy to treat refractory SSc and achieved more significant efficacy than second-generation CAR-T cells (1, 55). In the future, introducing pro-T cell cytokine genes, such as IL-12 and IL-15, may further enhance the effector function and *in vivo* expansion capacity of CAR-T cells (56).

Selecting SSc-specific antigens such as Scl-70 and centromere as new therapeutic targets may improve efficacy while reducing off-target toxicity. The CAAR-T strategy, by integrating autoantigens into the CAR, can specifically clear autoreactive B cells and may be applied to SSc patients with characteristic autoantibody positivity (47, 56).

Optimizing administration regimens, such as reducing fludarabine dosage in pretreatment and local administration, may lower the incidence and severity of CRS and neurotoxicity while ensuring CAR-T expansion (50, 57). For SSc with concomitant interstitial lung disease, bronchoscopic airway administration of CAR-T cells may enhance pulmonary T cell infiltration, directly inhibit pulmonary inflammation and fibrosis, and reduce systemic adverse reactions (56).

CAR-T cell and ADSC transplantation have brought new hope for the treatment of scleroderma. Fully leveraging the immunomodulatory and anti-fibrotic properties of both cell types and optimizing them through genetic engineering and tissue engineering techniques, may maximize the precision and effectiveness of cell therapy and extend the duration of its efficacy. In-depth research on scleroderma-specific cell therapy strategies, expansion of clinical research sample sizes, conduction of randomized controlled trials, and establishment of standardized efficacy evaluation and adverse reaction management systems are essential for advancing cell therapy into a routine precision treatment for scleroderma.

## 5 Challenges and countermeasures of cell therapy in scleroderma

### 5.1 Limitations and optimization strategies of key aspects such as cell preparation, administration routes, and efficacy evaluation

Cell preparation is key to ensuring therapeutic effects. Currently, the heterogeneity of mesenchymal stem cells (MSCs) is still a major challenge. MSCs derived from different sources exhibit significant differences in functions, such as immunomodulation (9, 11). Establishing a standardized quality control system and optimizing cell isolation, purification, and *in vitro* expansion are important ways to improve the efficacy of MSCs. In addition, the issue of cell viability loss during cryopreservation urgently needs to be addressed (58). Developing new cryoprotectants and advanced programmed freezing technologies may help maximize the retention of cell viability.

The choice of administration route directly affects therapeutic efficacy. Although intravenous infusion is simple to perform, the homing efficiency of cells *in vivo* remains low. While local injection can improve targeted delivery efficiency, it may not achieve systemic therapeutic effects. Developing new cell delivery systems, such as constructing injectable hydrogels in combination with biomaterials, may overcome the limitations of local administration. In addition, using cell-derived extracellular vesicles and other cell-free products for treatment can, to some extent avoid the safety risks of live cell infusion.

The lack of specific indicators for evaluating efficacy evaluation is another key issue. Currently, it mainly relies on clinical scores, such as mRSS and lacks biomarkers that can accurately reflect disease progression (59). Actively developing new molecular markers and imaging evaluation methods will yield more objective and accurate evidence for assessing the efficacy e of cell therapy. Discovering specific biomarkers through omics analysis and dynamically monitoring treatment responses using functional imaging are crucial directions for future development.

### 5.2 Combining cell therapy with conventional treatment methods to improve efficacy

The pathology of scleroderma is complex, and single treatment methods often fail to achieve satisfactory efficacy. Combining cell therapy with conventional treatment methods such as drugs may achieve a synergistic effect. For example, combining MSCs with immunosuppressants such as glucocorticoids may promote tissue repair while simultaneously controlling inflammation (5). Exploring the combined use of MSCs with vasoactive drugs may more effectively improve microvascular lesions (37). It is worth noting that biologics, such as anti-CD20 monoclonal antibodies, have shown good effects in scleroderma treatment (60). Combining these targeted drugs with cell therapy may achieve more precise immunomodulation. However, the safety and optimal timing for the administration of combination therapy regimens still need to be verified by rigorous clinical trials.

### 5.3 Functionally enhancing therapeutic cells targeting scleroderma pathogenesis to improve efficacy

Targeted modification of therapeutic cells for the specific pathological processes of scleroderma is a key strategy to improve efficacy. Using genetic engineering to make MSCs overexpress anti-fibrotic factors, such as HGF and KGF can significantly enhance their ability to inhibit fibrosis (61, 62). In addition, targeted regulation of signaling pathways, such as TGF-*β* can improve the therapeutic potential of MSCs within the scleroderma microenvironment (63). For CAR-T cell therapy, designing new CAR molecules, such as targeting specific antigens other than B cells may further expand its application in treating scleroderma (4, 55). However, the clinical safety and long-term effects of cell modification strategies still need to be carefully evaluated.

Using extracellular vesicles secreted by MSCs and other cells for treatment is another recently emerging research hotspot. Compared to live cells, cell-free products such as extracellular vesicles are more stable *in vivo* and may reduce safety risks, such as immunogenicity (64). Optimizing cell culture systems to selectively enrich specific miRNAs and other active components within extracellular vesicles could further enhance their immunomodulatory and anti-fibrotic functions. This strategy may improve the controllability of MSC therapy while retaining its therapeutic advantages.

### 5.4 Strengthening the evidence level of cell therapy clinical trials to support standardized clinical application

Several studies have preliminarily confirmed the efficacy and safety of cell therapy in scleroderma, but the current evidence level remains low. Most existing studies are small-sample, single-center, non-randomized controlled trials (14, 32). To achieve standardized application of cell therapy in scleroderma, high-quality prospective, multicenter randomized controlled clinical trials are still needed. At the same time, establishing standardized cell preparation and administration processes and optimizing dosage and administration frequency, is crucial for reducing the heterogeneity of clinical trial results (5, 65). Developing individualized cell therapy regimens tailored to different scleroderma subtypes and disease severities will be an important future direction.

In addition, the cost-effectiveness of cell therapy deserves attention. Currently, expenses associated with cell therapy are substantial, which may hinder patient accessibility (59). Actively exploring new technologies to improve cell preparation efficiency and reduce costs is crucial for promoting the widespread adoption of cell therapy (Table 1).

**Table 1:** CAR targeting various targets or MSC in different organs and systems affected by scleroderma.

Target/Action object	Effects	Related studies
CD19 for CAR-T	Improves skin sclerosis and lung function Reduces disease activity scores	Infusion of CD19 CAR-T cells from HLA-mismatched donors improved skin sclerosis and lung function in refractory SSc patients Phase I multicenter trials showed significant reduction in disease activity scores after a single infusion of CD19 CAR-T cells in SSc patients Genetically edited allogeneic CD19 CAR-T therapy improved organ function in refractory necrotizing myopathy and diffuse SSc patients
Scl70, centromere antigens (under exploration) for CAR-T	Enhances efficacy and reduces off-target toxicity Specifically eliminates autoreactive B cells Promising for SSc patients with characteristic autoantibodies	Research on CAAR-T strategies
MSC Therapy (Skin)	Reduces inflammatory infiltration and collagen deposition Improves sclerosis, elasticity, and appearance Promotes healing of refractory finger ulcers and relieves pain and dysfunction Autologous fat grafting improves texture and contour, promotes microcirculation reconstruction	Various MSCs reduced skin-related lesions in SSc mouse models Clinical studies showed reduced skin sclerosis after MSC infusion Studies on autologous fat grafting for treating facial skin sclerosis and finger ulcers
MSC Therapy (Lungs)	Reduces imbalance of Th1/Th2 cytokines Improves pulmonary fibrosis lesions Alleviates inflammation and delays fibrosis progression Improves pulmonary function and lowers pulmonary artery pressure	Animal model studies showed improvement in pulmonary fibrosis Research on bronchial injection of autologous fat-derived extracts for treating diffuse SSc with interstitial lung disease Clinical studies showed partial improvement in lung function
MSC Therapy (Blood vessels)	Promotes vascular regeneration Improves vascular damage, such as healing finger ulcers and reducing Raynaud’s phenomenon Enhances endothelial function and improves right heart function	Studies on MSC secretion of pro-angiogenic factors Clinical studies showed improvements in vascular lesions after MSC therapy
MSC Therapy (Immune System)	Suppresses proliferation and activation of immune cells Induces immune tolerance Reduces inflammatory cytokine levels and autoantibody titers	Studies on MSC secretion of immunosuppressive factors Clinical studies observed changes in inflammatory cytokines and autoantibodies after MSC therapy
MSC Therapy (Gastrointestinal Tract)	Alleviates gastrointestinal motility disorders and other visceral involvement	Observations from clinical studies

## 6 Summary and outlook

Scleroderma is a complex autoimmune disease involving multiple pathological processes, such as immune dysregulation, vascular damage, and tissue fibrosis. Although various treatment methods are currently available, they still fail to meet the clinical needs of scleroderma patients. With the development of precision medicine, cell therapy, as an emerging strategy, provides new possibilities for the precise treatment of scleroderma.

Mesenchymal stem cells (MSCs) and chimeric antigen receptor T cells (CAR-T) are two prominent representatives in the field of cell therapy. MSCs have multiple functions, including immunomodulation, anti-fibrosis, and promoting vascular regeneration, which may concurrently enhance various pathological processes of scleroderma. On the other hand, CAR-T cell therapy provides a new strategy for the targeted clearance of specific autoreactive immune cells, exhibiting stronger specificity and personalized potential.

In summary, cell therapy has opened new avenues for the precise treatment of scleroderma. In the future, it is essential to strengthen the integration of basic research and clinical application, establish standardized cell preparation and quality control, develop personalized treatment plans, and rationally combine cell therapy with existing treatment methods. These steps will maximize its advantages and improve the prognosis and quality of life of patients with scleroderma. It is believed that through the joint efforts of researchers and clinicians, cell therapy will undoubtedly promote the transformation of scleroderma treatment models and benefit a greater number of patients.

## References

[1] Müller F Taubmann J Bucci L Wilhelm A Bergmann C Völkl S. CD19 CAR T-cell therapy in autoimmune disease - a case series with follow-up. N Engl J Med. (2024) 390:687–700. doi: 10.1056/NEJMoa2308917, PMID: 38381673

[2] Khandpur S Gupta S Gunaabalaji DR. Stem cell therapy in dermatology. Indian J Dermatol Venereol Leprol. (2021) 87:753–67. doi: 10.25259/IJDVL_19_20, PMID: 34245532

[3] Wang X Wu X Tan B Zhu L Zhang Y Lin L. Allogeneic CD19-targeted CAR-T therapy in patients with severe myositis and systemic sclerosis. Cell. (2024) 187:4890–4904.e9. doi: 10.1016/j.cell.2024.06.027, PMID: 39013470

[4] Jones OY McCurdy D. Cell based treatment of autoimmune diseases in children. Front Pediatr. (2022) 10:855260. doi: 10.3389/fped.2022.855260, PMID: 35615628 PMC9124972

[5] Escobar-Soto C-H Mejia-Romero R Aguilera N Alzate-Granados JP Mendoza-Pinto C Munguía-Realpozo P. Human mesenchymal stem cells for the management of systemic sclerosis. Systematic review. Autoimmun Rev. (2021) 20:102831. doi: 10.1016/j.autrev.2021.102831, PMID: 33878487

[6] Peltzer J Aletti M Frescaline N Busson E Lataillade J-J Martinaud C. Mesenchymal stromal cells based therapy in systemic sclerosis: rational and challenges. Front Immunol. (2018) 9:2013. doi: 10.3389/fimmu.2018.02013, PMID: 30271402 PMC6146027

[7] Zhao K Kong C Shi N Jiang J Li P. Potential angiogenic, immunomodulatory, and antifibrotic effects of mesenchymal stem cell-derived extracellular vesicles in systemic sclerosis. Front Immunol. (2023) 14:1125257. doi: 10.3389/fimmu.2023.1125257, PMID: 37251412 PMC10213547

[8] Hagen M Wirsching A Bohr D Taubmann J Müller F Mackensen A. CAR T-cell therapy in rheumatology-what we know so far? Z Rheumatol. (2024) 83:485–91. doi: 10.1007/s00393-024-01514-x, PMID: 38780637 PMC11322323

[9] Xie L Long X Mo M Jiang J Zhang Q Long M. Bone marrow mesenchymal stem cell-derived exosomes alleviate skin fibrosis in systemic sclerosis by inhibiting the IL-33/ST2 axis via the delivery of microRNA-214. Mol Immunol. (2023) 157:146–57. doi: 10.1016/j.molimm.2023.03.017, PMID: 37028129

[10] Orvain C Boulch M Bousso P Allanore Y Avouac J. Is there a place for chimeric antigen receptor-T cells in the treatment of chronic autoimmune rheumatic diseases? Arthritis Rheum. (2021) 73:1954–65. doi: 10.1002/art.41812, PMID: 34042325

[11] Daumas A Magalon J Delaunay F Abellan M Philandrianos C Sabatier F. Fat grafting for treatment of facial scleroderma. Clin Plast Surg. (2020) 47:155–63. doi: 10.1016/j.cps.2019.08.016, PMID: 31739892

[12] Maria ATJ Toupet K Bony C Pirot N Vozenin M-C Petit B. Antifibrotic, antioxidant, and immunomodulatory effects of mesenchymal stem cells in HOCl-induced systemic sclerosis. Arthritis Rheum. (2016) 68:1013–25. doi: 10.1002/art.39477, PMID: 26474311

[13] Sierra-Sánchez Á Montero-Vilchez T Quiñones-Vico MI Sanchez-Diaz M Arias-Santiago S. Current advanced therapies based on human mesenchymal stem cells for skin diseases. Front Cell Dev Biol. (2021) 9:643125. doi: 10.3389/fcell.2021.643125, PMID: 33768095 PMC7985058

[14] Cras A Farge D Carmoi T Lataillade J-J Wang DD Sun L. Update on mesenchymal stem cell-based therapy in lupus and scleroderma. Arthritis Res Ther. (2015) 17:301. doi: 10.1186/s13075-015-0819-7, PMID: 26525582 PMC4631077

[15] Kuca-Warnawin E Olesińska M Szczȩsny P Kontny E. Impact and possible mechanism(s) of adipose tissue-derived mesenchymal stem cells on T-cell proliferation in patients with rheumatic disease. Front Physiol. (2021) 12:749481. doi: 10.3389/fphys.2021.749481, PMID: 35095547 PMC8793746

[16] Voswinkel J Francois S Simon J-M Benderitter M Gorin N-C Mohty M. Use of mesenchymal stem cells (MSC) in chronic inflammatory fistulizing and fibrotic diseases: a comprehensive review. Clin Rev Allergy Immunol. (2013) 45:180–92. doi: 10.1007/s12016-012-8347-6, PMID: 23296948

[17] Abdel Aziz MT Atta HM Mahfouz S Fouad HH Roshdy NK Ahmed HH. Therapeutic potential of bone marrow-derived mesenchymal stem cells on experimental liver fibrosis. Clin Biochem. (2007) 40:893–9. doi: 10.1016/j.clinbiochem.2007.04.017, PMID: 17543295

[18] Zanin-Silva DC Santana-Gonçalves M Kawashima-Vasconcelos MY Oliveira MC. Management of Endothelial Dysfunction in systemic sclerosis: current and developing strategies. Front Med (Lausanne). (2021) 8:788250. doi: 10.3389/fmed.2021.788250, PMID: 35004754 PMC8727451

[19] Rozier P Maumus M Maria ATJ Toupet K Jorgensen C Guilpain P. Lung fibrosis is improved by extracellular vesicles from IFNγ-primed mesenchymal stromal cells in murine systemic sclerosis. Cells. (2021) 10:2727. doi: 10.3390/cells10102727, PMID: 34685707 PMC8535048

[20] Maria ATJ Toupet K Maumus M Fonteneau G Le Quellec A Jorgensen C. Human adipose mesenchymal stem cells as potent anti-fibrosis therapy for systemic sclerosis. J Autoimmun. (2016) 70:31–9. doi: 10.1016/j.jaut.2016.03.013, PMID: 27052182

[21] Zhang Y Chopp M Liu XS Katakowski M Wang X Tian X. Exosomes derived from mesenchymal stromal cells promote axonal growth of cortical neurons. Mol Neurobiol. (2017) 54:2659–73. doi: 10.1007/s12035-016-9851-0, PMID: 26993303 PMC5028236

[22] Yamashita T Takahashi Y Takakura Y. Possibility of exosome-based therapeutics and challenges in production of exosomes eligible for therapeutic application. Biol Pharm Bull. (2018) 41:835–42. doi: 10.1248/bpb.b18-00133, PMID: 29863072

[23] Dudek DW Walczuk E Wajda A Paradowska-Gorycka A. Mesenchymal stem cells in systemic sclerosis therapy. Reumatologia. (2020) 58:324–30. doi: 10.5114/reum.2020.99995, PMID: 33227099 PMC7667949

[24] Wang HC Sun ET Zhao RC Chen B Han Q Li N. Adipose-derived stem cells attenuate skin fibrosis and improve fat retention of a localized scleroderma mouse model. Plast Reconstr Surg. (2023) 151:97–107. doi: 10.1097/PRS.0000000000009796, PMID: 36206077

[25] Zanin-Silva DC Santana-Gonçalves M Kawashima-Vasconcelos MY Lima-Júnior JR Dias JBE Moraes DA. Autologous hematopoietic stem cell transplantation promotes connective tissue remodeling in systemic sclerosis patients. Arthritis Res Ther. (2022) 24:95. doi: 10.1186/s13075-022-02779-w, PMID: 35488348 PMC9052524

[26] Wang H Sun E Zhao R Chen B Han Q Li N. Adipose-derived stem cells attenuate skin fibrosis and improve fat retention of a localized scleroderma mouse model. Plast Reconstr Surg. (2023) 1:97–107. 10.1097/PRS.0000000000009796 36206077

[27] Gong P Ding Y Sun R Jiang Z Li W Su X. Mesenchymal stem cells alleviate systemic sclerosis by inhibiting the recruitment of pathogenic macrophages. Cell Death Dis. (2022) 8:466. doi: 10.1038/s41420-022-01264-2, PMID: 36435837 PMC9701228

[28] Liang J Zhang H Kong W Deng W Wang D Feng X. Safety analysis in patients with autoimmune disease receiving allogeneic mesenchymal stem cells infusion: a long-term retrospective study. Stem Cell Res Ther. (2018) 9:312. doi: 10.1186/s13287-018-1053-4, PMID: 30428931 PMC6236873

[29] Zhang H Liang J Tang X Wang D Feng X Wang F. Sustained benefit from combined plasmapheresis and allogeneic mesenchymal stem cells transplantation therapy in systemic sclerosis. Arthritis Res Ther. (2017) 19:165. doi: 10.1186/s13075-017-1373-2, PMID: 28724445 PMC5518166

[30] Suzuka T Kotani T Saito T Matsuda S Sato T Takeuchi T. Therapeutic effects of adipose-derived mesenchymal stem/stromal cells with enhanced migration ability and hepatocyte growth factor secretion by low-molecular-weight heparin treatment in bleomycin-induced mouse models of systemic sclerosis. Arthritis Res Ther. (2022) 24:228. doi: 10.1186/s13075-022-02915-6, PMID: 36207753 PMC9540693

[31] Molberg Ø Hoffmann-Vold A-M. Interstitial lung disease in systemic sclerosis: progress in screening and early diagnosis. Curr Opin Rheumatol. (2016) 28:613–8. doi: 10.1097/BOR.0000000000000323, PMID: 27387267

[32] Guiducci S Manetti M Romano E Mazzanti B Ceccarelli C Dal Pozzo S. Bone marrow-derived mesenchymal stem cells from early diffuse systemic sclerosis exhibit a paracrine machinery and stimulate angiogenesis in vitro. Ann Rheum Dis. (2011) 70:2011–21. doi: 10.1136/ard.2011.150607, PMID: 21821866

[33] Lee R Del Papa N Introna M Reese CF Zemskova M Bonner M. Adipose-derived mesenchymal stromal/stem cells in systemic sclerosis: alterations in function and beneficial effect on lung fibrosis are regulated by caveolin-1. J Scleroderma Relat Disord. (2019) 4:127–36. doi: 10.1177/2397198318821510, PMID: 35382388 PMC8922642

[34] Li M Zhang H-P Wang X-Y Chen Z-G Lin X-F Zhu W. Mesenchymal stem cell-derived exosomes ameliorate dermal fibrosis in a murine model of bleomycin-induced scleroderma. Stem Cells Dev. (2021) 30:981–90. doi: 10.1089/scd.2021.0112, PMID: 34428952

[35] Ramalingam S Shah A. Stem cell therapy as a treatment for autoimmune disease-updates in lupus, scleroderma, and multiple sclerosis. Curr Allergy Asthma Rep. (2021) 21:22. doi: 10.1007/s11882-021-00996-y, PMID: 33759038

[36] Alip M Wang D Zhao S Li S Zhang D Duan X. Umbilical cord mesenchymal stem cells transplantation in patients with systemic sclerosis: a 5-year follow-up study. Clin Rheumatol. (2024) 43:1073–82. doi: 10.1007/s10067-024-06865-z, PMID: 38206544

[37] Kim KH Blasco-Morente G Cuende N Arias-Santiago S. Mesenchymal stromal cells: properties and role in management of cutaneous diseases. J Eur Acad Dermatol Venereol. (2017) 31:414–23. doi: 10.1111/jdv.13934, PMID: 27549663

[38] AlOdhaibi KA Varga J Furst DE. Hematopoietic stem cell transplantation in systemic sclerosis: yes!! BUT J Scleroderma Relat Disord. (2021) 6:44–9. doi: 10.1177/2397198320971967, PMID: 35382253 PMC8922628

[39] Shi Y Jiang N Li M Zeng X Tian X. Mesenchymal stem cells and connective tissue diseases: from bench to bedside. J Transl Int Med. (2023) 11:30–45. doi: 10.2478/jtim-2022-0028, PMID: 37533846 PMC10393058

[40] Maria ATJ Maumus M Le Quellec A Jorgensen C Noël D Guilpain P. Adipose-derived mesenchymal stem cells in autoimmune disorders: state of the art and perspectives for systemic sclerosis. Clin Rev Allergy Immunol. (2017) 52:234–59. doi: 10.1007/s12016-016-8552-9, PMID: 27207172

[41] Rozier P Maria A Goulabchand R Jorgensen C Guilpain P Noël D. Mesenchymal stem cells in systemic sclerosis: allogenic or autologous approaches for therapeutic use? Front Immunol. (2018) 9:2938. doi: 10.3389/fimmu.2018.02938, PMID: 30619298 PMC6302042

[42] Jinnin M Ihn H Yamane K Tamaki K. Interleukin-13 stimulates the transcription of the human alpha2(I) collagen gene in human dermal fibroblasts. J Biol Chem. (2004) 279:41783–91. doi: 10.1074/jbc.M406951200, PMID: 15271999

[43] Keyßer G. Mesenchymal stem cell treatment in autoimmune diseases. Z Rheumatol. (2020) 79:437–45. doi: 10.1007/s00393-020-00790-7, PMID: 32322976

[44] Lansiaux P Farge D. Contribution of mesenchymal stromal cell transplantation in systemic scleroderma. Rev Prat. (2022) 72:355–62. 35638974

[45] Magalon G Daumas A Sautereau N Magalon J Sabatier F Granel B. Regenerative approach to scleroderma with fat grafting. Clin Plast Surg. (2015) 42:353–64. doi: 10.1016/j.cps.2015.03.009, PMID: 26116941

[46] Lee B-W Kwok S-K. Mesenchymal stem/stromal cell-based therapies in systemic rheumatic disease: from challenges to new approaches for overcoming restrictions. Int J Mol Sci. (2023) 24:10161. doi: 10.3390/ijms241210161, PMID: 37373308 PMC10299481

[47] Yang Y Jacoby E Fry TJ. Challenges and opportunities of allogeneic donor-derived CAR T cells. Curr Opin Hematol. (2015) 22:509–15. doi: 10.1097/MOH.0000000000000181, PMID: 26390167 PMC4636084

[48] Toubai T Sun Y Reddy P. GVHD pathophysiology: is acute different from chronic? Best Pract Res Clin Haematol. (2008) 21:101–17. doi: 10.1016/j.beha.2008.02.005, PMID: 18503979

[49] Shah NN Fry TJ. Mechanisms of resistance to CAR T cell therapy. Nat Rev Clin Oncol. (2019) 16:372–85. doi: 10.1038/s41571-019-0184-6, PMID: 30837712 PMC8214555

[50] Rong X Xia X Wang R Su Z Liu T Zhang Z. Near-infrared and highly photostable squaraine-based nanoparticles for photoacoustic imaging guided photothermal therapy. Dyes Pigments. (2023) 211:111055. doi: 10.1016/j.dyepig.2022.111055

[51] Chalayer E Gramont B Zekre F Goguyer-Deschaumes R Waeckel L Grange L. Fc receptors gone wrong: a comprehensive review of their roles in autoimmune and inflammatory diseases. Autoimmun Rev. (2022) 21:103016. doi: 10.1016/j.autrev.2021.103016, PMID: 34915182

[52] Bergmann C Müller F Distler JHW Györfi A-H Völkl S Aigner M. Treatment of a patient with severe systemic sclerosis (SSc) using CD19-targeted CAR T cells. Ann Rheum Dis. (2023) 82:1117–20. doi: 10.1136/ard-2023-223952, PMID: 37147112 PMC10359520

[53] Merkt W Freitag M Claus M Kolb P Falcone V Röhrich M. Third-generation CD19.CAR-T cell-containing combination therapy in Scl70+ systemic sclerosis. Ann Rheum Dis. (2024) 83:543–6. doi: 10.1136/ard-2023-225174, PMID: 38135464 PMC10958299

[54] Chasov V Zmievskaya E Ganeeva I Gilyazova E Davletshin D Khaliulin M. Immunotherapy strategy for systemic autoimmune diseases: betting on CAR-T cells and antibodies. Antibodies (Basel). (2024) 13:10. doi: 10.3390/antib13010010, PMID: 38390871 PMC10885098

[55] Lyu X Gupta L Tholouli E Chinoy H. Chimeric antigen receptor T cell therapy: a new emerging landscape in autoimmune rheumatic diseases. Rheumatology (Oxford). (2024) 63:1206–16. doi: 10.1093/rheumatology/kead616, PMID: 37982747 PMC11065442

[56] Khanna D Krieger N Sullivan KM. Improving outcomes in scleroderma: recent progress of cell-based therapies. Rheumatology (Oxford). (2023) 62:2060–9. doi: 10.1093/rheumatology/keac628, PMID: 36355455 PMC10234204

[57] Xie F Xu M Lu J Mao L Wang S. The role of exosomal PD-L1 in tumor progression and immunotherapy. Mol Cancer. (2019) 18:146. doi: 10.1186/s12943-019-1074-3, PMID: 31647023 PMC6813045

[58] Ohno R Nakamura A. Advancing autoimmune rheumatic disease treatment: CAR-T cell therapies - evidence, safety, and future directions. Semin Arthritis Rheum. (2024) 67:152479. doi: 10.1016/j.semarthrit.2024.152479, PMID: 38810569

[59] Tyndall A Furst DE. Adult stem cell treatment of scleroderma. Curr Opin Rheumatol. (2007) 19:604–10. doi: 10.1097/BOR.0b013e3282e6f534, PMID: 17917542

[60] Rubbert-Roth A Furst DE Nebesky JM Jin A Berber E. A review of recent advances using tocilizumab in the treatment of rheumatic diseases. Rheumatol Ther. (2018) 5:21–42. doi: 10.1007/s40744-018-0102-x, PMID: 29502236 PMC5935615

[61] Allan GJ Beattie J Flint DJ. Epithelial injury induces an innate repair mechanism linked to cellular senescence and fibrosis involving IGF-binding protein-5. J Endocrinol. (2008) 199:155–64. doi: 10.1677/JOE-08-0269, PMID: 18676497

[62] Spierings J van Laar JM. Is there a place for hematopoietic stem cell transplantation in rheumatology? Rheum Dis Clin N Am. (2019) 45:399–416. doi: 10.1016/j.rdc.2019.04.003, PMID: 31277752

[63] Vanneaux V Farge-Bancel D Lecourt S Baraut J Cras A Jean-Louis F. Expression of transforming growth factor β receptor II in mesenchymal stem cells from systemic sclerosis patients. BMJ Open. (2013) 3:e001890. doi: 10.1136/bmjopen-2012-001890, PMID: 23299111 PMC3549232

[64] Swain HN Boyce PD Bromet BA Barozinksy K Hance L Shields D. Mesenchymal stem cells in autoimmune disease: a systematic review and meta-analysis of pre-clinical studies. Biochimie. (2024) 223:54–73. doi: 10.1016/j.biochi.2024.04.009, PMID: 38657832

[65] Arakkal G Chintagunta SR Chandika V Damarla SV Manchala S Kumar BU. Cardio-pulmonary involvement in systemic sclerosis: a study at a tertiary care center. Indian J Dermatol Venereol Leprol. (2017) 83:677–82. doi: 10.4103/0378-6323.198453, PMID: 29035287

